# Social and Physical Environments and Self-Rated Health in Urban and Rural Communities in Korea

**DOI:** 10.3390/ijerph121114329

**Published:** 2015-11-12

**Authors:** Jung-A Lee, Jong Heon Park, Myung Kim

**Affiliations:** 1 Department of Health Management, Graduate School, Ewha Womans University, Seoul 120-750, Korea; E-Mail: leejabelle@gmail.com; 2 Department of Health and Medical Information, Myongji College, Seoul 120-776, Korea; 3 Department of Big Data Steering, National Health Insurance Service, Seoul 121-749, Korea; E-Mail: jmhoney0@gmail.com; 4 Department of Health Education and Management, College of Health Sciences, Ewha Womans University, Seoul 120-750, Korea

**Keywords:** social and physical environment, SRH, urban, rural

## Abstract

This study evaluated the associations between social and physical environments and self-rated health (SRH) for urban and rural Korean adults, using data from the Korean Community Health Survey (KCHS) of 199,790 participants (115,454 urban and 84,336 rural). The main dependent variable was SRH, while the primary independent variables were social and physical characteristics. Urban residents reported better SRH than did rural residents. Five social environmental variables (trust of neighbors, residence in the area for over 20 years, exchanging help with neighbors, friend and fellowship activities, contact with relatives and neighbors over five times per month) were more prevalent among rural residents. Satisfaction with physical environment was more common among rural residents, but satisfaction with traffic and healthcare facilities was more common among urban areas. After adjusting for relevant factors, positive associations between SRH and trust of neighbors, exchanging help with neighbors, participation in social activities or organizations, and physical environment existed in both rural and urban populations. Also, in both areas, there was no demonstrated association between SRH and years of residence or frequency of contact with relatives. Our findings suggest the existence of an association between social and physical factors and perceived health status among the general population of Korea.

## 1. Introduction

A local community is more than a population with various risk factors for disease; rather, it influences health through social and physical surroundings, that is, environments [1]. Social environments include social relationships, social support, and social norms, while physical environmental factors include parks, buildings, noise pollution, traffic, among others. Community-based health programs have focused on the impact of individual health behaviors and therefore placed more emphasis on health education, which is aimed at improving individual health behaviors and forming healthy lifestyle habits [2]. However, the same degree of improvement in health status has not occurred in every social group, and there are still significant differences in health levels between local areas. For this reason, studies have suggested that health education programs and other projects must be tailored to the local area’s social or environmental characteristics [3,4]. According to these studies, differences in individual health derive from interactions between the physical or social environments of the community and individual health behaviors, medical services, or genetic factors. Since both physical and social environmental factors have been found to significantly influence local population health, it would be meaningful to develop health promotion programs focusing on a community’s social and physical environmental characteristics. 

Previous studies have used proxy variables to represent the social and physical environments rather than measuring the social and physical environments as perceived by the individual. For social environment, these factors include the number of consultative groups, frequency of holding conferences, and others; for physical environment, proxy variables include the number of social enterprises, facilities for the disabled, the size of local parks, the ratio of roads, and others [5,6]. This enables group-level comparisons between regions or countries.

However, there is some debate as to whether social and physical environments can be adequately captured by such proxy variables [4,7,8]. If proxy variables are used to approximate the influence of the local social environment on the individual, it might lead to conclusions that ignore the differences between individuals that affect individual health, since individuals of a local area will perceive social environments differently [9]. In a study by Poortinga *et al.* individual levels of social trust and social activity were strongly associated with self-rated health (SRH) [8]. 

On the other hand, social ecological model is applied as effective theoretical framework to explain factors, which has efficiency to health and health behavior. Ecological model are believed to provide comprehensive frameworks for understanding the multiple and interacting determinants of health behaviors. Behavior change is expected to be maximized when environments and policies support healthful choices, when social norms and social support for healthful choices are strong, and when individuals are motivated and educated to make those choices. With this logic, we investigated the relationships between social and physical environments and SRH. Conceptual framework for variable and analysis on research are selected from contents covered in Kobayashi’s research that evaluated the association between social capital measured by the Resource Generator and SRH among the Japanese population [10,11].

According to prior studies, in local areas with better social support and strongly bonded community members, these features have a positive impact on health behavior and consequently increase overall health status [5,12,13]. Similarly, it has been suggested that physical environmental factors such as accessibility of health exercise facilities, bike lanes, and trails can influence individual tendencies towards obesity, heart disease, diabetes, and respiratory diseases [14,15,16]. Nevertheless, relatively few comparative studies of urban and rural area have examined the association of social capital and area (urban and rural) or individual characteristics [17,18]. An individual’s perception of their social and physical environment can differ by country or region, due to their own social and cultural difference. These differences can affect individual health status. Social environments enables or constrains the adoption of health promoting behavior, provides access to resources and material goods, provides individual and community coping responses, buffers negative health outcomes. Also it has been reported in several studies that social environments such as trust, associational membership, and reciprocity are influencing self-rated health. Accordingly, in this study, we investigated the relationships between social and physical environments and SRH (as health related outcome variable) in urban and rural Korea. There are numerous definitions of community [19]. Communities have been defined as functional spatial units meeting basic needs for sustenance, units of patterned social interaction, symbolic units of collective identity, and social unit where people come together politically to make changes [19]. The ecological system perspective is particularly useful in the study of autonomous geographical communities, focusing as it does on population characteristics such as size, density, and heterogeneity, the physical environment, the social organization or structure of the community, and the technological forces affecting it [19]. In this study community have been defined as spatial units using administrative areas (Si-Gun-Gu).

We add variable of residence years as social environment. Although years of residence do not effect health status directly, however, if longer year in community, that could be explained as community with same experience. It signifies that community has a tendency to trust each other that people are organically and closely connected. The sense of community and fellowship and trustworthy lead to people to participate in society more equally. Also, it is easy to have social safety and health, and it increases accessibility to share and spread information, and handle informal health behavior-smoking and drinking from affective support. 

## 2. Methods

### 2.1. Databases

Data were obtained from the Korean Community Health Survey (KCHS) in 2013 [20]. KCHS is a nationwide survey that has been conducted annually since 2008. This community-based cross-sectional survey has been conducted in 16 metropolitan cities and provinces with 253 regional sites. KCHS covered a wide variety of health topics, which can be used to assess the prevalence of personal health behaviors related to the causes of disease. The survey was conducted by trained interviewers as a one-to-one interview based on a protocol and questionnaires. The Community Health Survey was conducted in 800–900 subjects selected by a standardized sampling method of adults aged 19 years or older living in each area from August to October 2013. We selected 199,790 of the 228,781 participants in the 2013 KCHS and used data on social and physical environmental factors: social participation and activities, network connections, trust of neighbors, and satisfaction with the physical environment. Smallest administrative district units in Si-Gun-Gu was selected as the primary sampling unit of housing types in Dong/Eup/Myeon. The primary sample units were used in census, which is regarded as the biggest national survey in Korea. Health survey handled by local unit also used the same units, even for the biggest Korea Community Health Survey. The KCHS survey was approved by the Institutional Review Board of the Korea Centers for Disease Control and Prevention. Written consent was obtained from the participants.

### 2.2. Definition of Variables

The survey instrument included 15 items that assessed the social and physical environments using five indicators: trust of neighbors, years of residence, exchanging help with neighbors, social activities and participation, perceived network connections, and satisfaction with local area. Trust of and exchanging help with neighbors were each assessed by one item: “People in this neighborhood can be trusted” (yes/no); and “People in the community help their neighbors with family occasions/events” (yes/no), respectively. ‘Exchanging help with neighbors’ should be understood in the context of Korean traditional culture, in which people gather together for good and bad occasions and often exchange money for special occasions such as weddings or funerals. In rural areas, people also often support others in need by giving money or buying goods. Period of residence was covered by one question: “How long have you lived in your community?” (under 5 years, 5–9 years, 10–14 years, 15–19 years, over 20 years). The social activities and participation category was assessed by four questions regarding participation in religious organizations, friend and fellowship activities, recreational and leisure groups/organizations, and volunteer work/activities (yes/no). Zunzunegui *et al.* showed that social networks are defined by their structure and function (frequency of contact) and can be further classified into subnetworks according to the nature or specific role of the relationship in question (relatives, neighbors, friends) [21]. In this study, social network was covered by three questions: “How often do you speak or meet up with relatives/neighbors/friends who are not living with you?” (less than once per month, 2–4 times per month, over 5 times per month). Perceived local area satisfaction was assessed by five items, covering factors regarding which participants were asked if they were satisfied with their local area as a place to live: “Overall safety (such as crime levels, local services)”, “Natural environment (such as water, air, parks, pollution)”, “Living environment (such as electronic, trash/rubbish collection service, exercise facilities)”, “Traffic conditions”, “Access to health services” (yes/no). 

Our outcome measure was SRH measured by a single questionnaire item: “How would you describe your overall state of health?” (excellent, good, medium, poor, very poor). For use as an outcome measure, the item was dichotomized as “good” (excellent or good) or “poor” (medium, poor, or very poor). Measures of health status and behavior included self-reported weight (kg) and height (cm), smoking status (none/never and current), frequency of alcohol use (none/never, sometimes, often), and frequency of physical activity (none, sometimes, often). Age and body mass index (BMI) were continuous variables, drawn from the KCHS. Education status was assessed by four categories (below elementary school, middle school, high school, and college or above). Areas were categorized as urban or rural according to administrative district. The final variable was living arrangement (living with others, living alone) [11]. 

### 2.3. Statistical Analyses

Descriptive analyses were performed to clarify the distribution of participant demographics. We compared continuous and dichotomized categorical data between urban and rural residence populations. We used log binomial regression to examine the relationships between social and physical environmental factors and outcome, after adjusting for demographic information (age, gender, BMI, education, living arrangement, smoking and alcohol status, frequency of physical activity). Controlling for all other variables in the regression analyses, prevalence ratios (PRs) with 95% confidence intervals and *p* values < 0.05 were considered statistically significant. Data analyses were performed using the SAS statistical software (ver. 9.3).

## 3. Results

### 3.1. Characteristics of Urban and Rural Participants

Table 1 shows study subject characteristics by area (115,454 urban and 84,336 rural). The mean age was 47.5 years for urban and 56.2 years for rural area residents. There were greater proportions of women than men in both areas. The proportion of people who were educated at the college or higher level was greater in urban areas, whereas the proportion of people educated below the elementary school level was greater in rural areas. Urban participants were more likely to have good or very good SRH compared with rural participants (41.7% *vs.* 37.0%). The distributions of other sociodemographic characteristics were similar between urban and rural residents. The percentage of respondents in each category is shown for categorical variables, while means and standard deviations are shown for continuous variables.

**Table 1:** Sociodemographic characteristics of the study population according to area.

Variables	Urban (N = 115,454)	Rural (N = 84,336)	*p* *
	N (%)	N (%)	
Age (years; mean, SD)	47.5 (16.0)	56.2 (16.1)	<0.001
Gender			
Male	52,560 (45.5)	40,467 (48.0)	<0.001
Female	62,894 (54.5)	43,869 (52.0)	
BMI (kg/m^2^; Mean, SD)	23.1 (3.1)	23.1 (3.1)	<0.01
Education attainment			
Elementary	16,992 (14.7)	32,885 (39.0)	<0.001
Middle	11,543 (10.0)	11,738 (13.9)	
High	37,291 (32.3)	23,285 (27.6)	
College or more	49,628 (43.0)	16,428 (19.5)	
Living arrangements			
Living with others	105,996 (91.8)	74,456 (88.3)	<0.001
Living alone	9458 (8.2)	9880 (11.7)	
Smoking			
Never/former	91,225 (79.0)	67,156 (79.6)	<0.01
Current	24,229 (21.0)	17,180 (20.4)	
Alcohol ^a^			
None/never	49,742 (43.1)	44,983 (53.3)	<0.001
Sometimes	40,733 (35.3)	20,472 (24.3)	
Often	24,979 (21.6)	18,881 (22.4)	
Physical activity ^b^			
None	8689 (7.5)	13,313 (15.8)	<0.001
Sometimes	11,444 (9.9)	7678 (9.1)	
Often	95,321 (82.6)	63,345 (75.1)	
SRH			
Good	48,184 (41.7)	31,246 (37.0)	<0.001
Poor	67,270 (58.3)	53,090 (63.0)	

Notes: SD = standard deviation; BMI = body mass index. * Significance according to t-test or chi-square test between urban and rural areas. ^a^ Categorized as follows: none/never (less than 1 day per month), sometimes (1 day per month to 2~4 days per month), often (2 or 3 days per week to over 4 days per week); ^b^ Categorized as follows: none (less than 1 day per week), sometimes (1 day per week to 2 days per week), often (3 days per week to over 5 days per week).

### 3.2. Characteristics of Social and Physical Environment according to Area

The social and physical environmental characteristics by area are shown in Table 2. Fifty-nine percent of urban residents agreed that people in their neighborhood could be trusted, as compared with 80.8% of rural residents. In both areas, the greatest proportion of people had resided in the area for over 20 years. The proportion of exchanges of help with neighbors was greater in rural areas (80.2% *vs.* 35.1%), and that of participation in leisure groups or organizations was greater in urban areas (32.1% *vs.* 20.5%). The large proportion of urban residents had contact with family members less than once a month, but the high proportion of rural residents had contact with family members more than five times a month. For both areas, the proportion of residents had higher contact with friends over five times per month than under five times per month. A majority of rural residents were satisfied with their physical environment in terms of overall safety, natural environment, and living conditions, but not in terms of traffic or healthcare access. 

**Table 2:** Social and physical environmental factors according to area.

Variables	Urban (N = 115,454)	Rural (N = 84,336)	*p* *
	N (%)	N (%)	
*Social environment*			
Trust neighbors (yes)	67,965 (58.9)	68,146 (80.8)	<0.001
Years of residence			
Under 5 years	18,011 (15.6)	7787 (9.2)	<0.001
5–9 years	13,684 (11.9)	6138 (7.3)	
10–14 years	13,079 (11.3)	4976 (5.9)	
15–19 years	10,428 (9.0)	4252 (5.0)	
Over 20 years	60,252 (52.2)	61,183 (72.5)	
Exchange of help with neighbors (yes)	40,524 (35.1)	67,642 (80.2)	<0.001
Religious participation (yes)	34,239 (29.7)	22,136 (26.2)	<0.001
Friend and fellowship activities (yes)	67,336 (58.3)	50,124 (59.4)	<0.001
Leisure groups/organizations (yes)	37,058 (32.1)	17,307 (20.5)	<0.001
Volunteer work/activities (yes)	9749 (8.4)	6465 (7.7)	<0.001
Frequency of contact with relatives			
Under once/month	42,571 (36.9)	22,470 (26.6)	<0.001
2~4 times/month	35,444 (30.7)	25,273 (30.0)	
Over 5 times/month	37,439 (32.4)	36,593 (43.4)	
Frequency of contact with neighbors			
Under once/month	58,843 (51.0)	13,076 (15.5)	<0.001
2~4 times per month	19,246 (16.7)	11,046 (13.1)	
Over 5 times/month	37,365 (32.4)	60,214 (71.4)	
Frequency of friends contact			
Under once/month	35,286(30.6)	31,386 (37.2)	<0.001
2~4 times/month	32,181 (27.9)	18,814 (22.3)	
Over 5 times/month	47,987 (41.6)	34,136 (40.5)	
*Physical environment satisfaction*			
Overall safety (yes)	82,369 (71.3)	72,404 (85.9)	<0.001
Natural (yes)	86,873 (75.2)	74,561 (88.4)	<0.001
Living (yes)	90,522 (78.4)	67,143 (79.6)	<0.001
Traffic (yes)	88,042 (76.3)	48,162 (57.1)	<0.001
Health services access (yes)	82,753 (71.7)	47,976 (56.9)	<0.001

Note: * Significance according to chi-square test between urban and rural areas.

### 3.3. Association of SRH and Social and Physical Environment

Prevalence ratios (PRs) were examining the predictive power of social and physical environment on poor SRH and reported in Table 3. In both areas, adjusted PRs significance was noted in the following social environmental variables: mistrust of neighbors, people who did not engage in exchange of help with neighbors, those who did not participated in religious organizations, those who did not participate in friend or fellowship activities or in leisure groups/organizations, those who did not participate in volunteer work/activities. 

**Table 3:** Prevalence ratios (PRs) of social and physical environment factors and poor SRH.

Variables	Urban				Rural			
	Crude		Adjusted		Crude		Adjusted	
	PR	95% CI	PR	95% CI	PR	95% CI	PR	95% CI
Social environment								
Trust of neighbors (no)	1.04	1.03–1.05	1.12	1.11–1.13	0.97	0.96–0.99	1.15	1.14–1.27
Years of residence								
Under 5 years	1.00		1.00		1.00		1.00	
5–9 years	1.05	1.03–1.07	1.00	0.99–1.02	1.03	1.00–1.06	0.99	0.96–1.02
10–14 years	1.04	1.02–1.06	0.99	0.97–1.01	1.03	0.99–1.06	0.97	0.94–1.00
15–19 years	1.00	0.97–1.02	0.97	0.95–0.99	0.97	0.93–1.01	0.96	0.93–1.00
Over 20 years	1.14	1.12–1.16	0.97	0.96–0.99	1.27	1.24–1.30	0.99	0.96–1.01
Exchange of help with neighbors (no)	0.95	0.94–0.96	1.08	1.07–1.09	0.90	0.89–0.91	1.12	1.10–1.13
Religious participation (no)	0.96	0.95–0.97	1.05	1.04–1.06	1.00	0.99–1.01	1.04	1.02–1.05
Friend and fellowship activities (no)	1.09	1.08–1.10	1.12	1.11–1.13	1.18	1.17–1.19	1.10	1.09–1.11
Leisure groups/organizations (no)	1.33	1.31–1.34	1.20	1.19–1.21	1.41	1.39–1.43	1.15	1.13–1.17
Volunteer work/activities (no)	1.17	1.14–1.19	1.14	1.12–1.16	1.28	1.24–1.31	1.13	1.10–1.16
Frequency of relatives contact								
Under once/month	1.00		1.00		1.00		1.00	
2~4 times/month	1.01	1.00–1.02	0.98	0.97–0.99	1.00	0.99–1.01	0.99	0.97–1.00
Over 5 times/month	1.02	1.01–1.03	0.97	0.96–0.98	1.00	0.99–1.02	0.97	0.96–0.98
Frequency of neighbors contact								
Under once/month	1.00		1.00		1.00		1.00	
2~4 times/month	1.03	1.02–1.05	0.96	0.94–0.97	1.02	1.00–1.05	0.91	0.90–0.93
Over 5 times/month	1.13	1.12–1.14	0.94	0.93–0.95	1.16	1.14–1.18	0.89	0.87–0.90
Frequency of friends contact								
Under once/month	1.00		1.00		1.00		1.00	
2~4 times/month	0.85	0.84–0.86	0.94	0.93–0.95	0.83	0.82–0.85	0.96	0.94–0.97
Over 5 times/month	0.80	0.79–0.81	0.89	0.88–0.90	0.82	0.81–0.83	0.93	0.92–0.94
Physical environment satisfaction								
Overall safety (no)	1.09	1.08–1.10	1.13	1.12–1.14	1.02	1.00–1.03	1.16	1.14–1.18
Natural (no)	1.09	1.08–1.10	1.12	1.11–1.13	1.03	1.01–1.04	1.11	1.09–1.12
Living (no)	1.08	1.07–1.09	1.12	1.11–1.13	1.00	0.98–1.01	1.09	1.07–1.10
Traffic (no)	1.04	1.03–1.05	1.07	1.06–1.08	0.99	0.98–1.00	1.05	1.04–1.06
Healthcare facilities (no)	1.06	1.04–1.07	1.10	1.08–1.11	0.98	0.97–0.99	1.06	1.05–1.07

Notes: PR = prevalence ratio; CI = confidence interval; Adjusted for age, gender, BMI, education attainment, living arrangements, smoking, alcohol and physical activity; self-rated health categorized as poor health (medium/poor/very poor) and better health (very good/good).

The variable of residence duration seemed to be associated with poor SRH in the crude model, however, it was not significant statistically in the adjusted model in both areas. In both areas, SRH was significantly poorer for those who were in contact with relatives under once per month than over five times per month, and being in contact with neighbors under once per month than more than twice per month (2–4 times per month: urban adjusted PR = 0.96, 95% CI: 0.94–0.97, rural adjusted PR = 0.91, 95% CI: 0.90–0.93; over five times per month: urban adjusted PR = 0.94, 95% CI: 0.93–0.95, rural adjusted PR = 0.89, 95% CI: 0.87–0.90), and being in contact with friends under once per month was associated with poor health in comparison with more than twice per month (2–4 times per month: urban adjusted PR = 0.94, 95% CI: 0.93–0.95; rural adjusted PR = 0.96, 95% CI: 0.94–0.97, over five times per month: urban adjusted PR = 0.89, 95% CI: 0.88–0.90; rural adjusted PR = 0.93, 95% CI: 0.92–0.94).

In terms of physical environmental characteristics, in both areas, SRH was significantly poorer in those who were not satisfied with overall safety, their natural and living environment, traffic conditions, and healthcare facilities than in those who were satisfied, after adjusting for age, gender, BMI, education attainment, living arrangements, smoking and alcohol status, and physical activity.

## 4. Discussion

This study analyzed the associations between perceived social or physical environmental characteristics and SRH among individual members of urban and rural populations in Korea. We found a positive association between a more favorable social environment (trust of neighbors; exchanging help with neighbors; participation in religious organizations, friend and fellowship activities, leisure groups/organizations, and volunteer work/activities; more contact with friends) and SRH in both urban and rural areas after covariate adjustment. This supports findings from previous nationwide studies performed in other countries. Greiner *et al.* showed a consistent association between a greater involvement in the community and better individual SRH and behaviors by area in Kansas (USA) [17]. Poortinga similarly demonstrated in 22 European countries that those with higher levels of trust and civic participation were more likely to report good health results [8]. Zunzunegui *et al.* showed a consistent association between social relationships and SRH among older individuals in two French-speaking Canadian communities [21]. Nummela *et al.* showed consistently high levels of social participation and trust among older individuals in Finland, suggesting that better health, which enabled them to live longer, healthier lives, was associated with a better social environment [22]. Ziersch *et al.* showed that higher levels of trust, cohesion, and help were associated with better mental health in rural and urban communities in South Australia [23], and Yip *et al.* used multilevel analysis to show that trust and reciprocity were positively associated with self-reported general health, psychological health, and subjective well-being in rural China [24]. 

In rural areas, there is an inverse association between a longer term of residence—particularly that over 20 years—and poor health. We initially anticipated that participants who had resided longer in their area would have better SRH than others. Previous studies have reported associations between longer term of residence and higher social participation and cooperation in the local area. In our study, considering that trust of neighbors, exchanging help with neighbors, participation in religious organizations, friend and fellowship activities, leisure groups/organizations, volunteer work/activities, and contact with friends were found to be predictors of better SRH, duration of residence is also expected to be a contributing factor. However, we found that health status in those with longer residence terms was as poor as or poorer than that in those with shorter residence terms in rural areas, even after adjusting for potential confounders. This is related to the fact that many people do not participate or cooperate within their local community, even when they have been living there for many years. We suspect that while long-term residence is a factor in an individual’s degree of participation and cooperation in the local community, it has an indirect rather than a direct impact on individual health status. 

We found that the impact of physical environmental factors such as safety, natural and living conditions, traffic, and health service facilities on individual SRH. Consistent with the literature, positive physical characteristics were positively associated with perceived health and health behaviors [25,26,27,28,29]. That is, a high level of satisfaction with their community environment (such as natural and living conditions, safety, traffic, healthcare facilities) may improve the residents to engage in better health outcome. In our study, urban residents had slightly better SRH than that of rural residents. Rural residents were more likely to report satisfaction with overall safety, natural environment, and living conditions, while urban residents were more likely to report satisfaction with traffic and access to healthcare services. Rural populations have the advantages of a cleaner atmosphere and access to nature, arguably giving them more opportunity to engage in healthy behaviors. However, urban populations have greater access to healthcare services and health information, arguably giving them greater control over their own health. 

This study had some limitations. First, we did not validate the questions in the self-reported social and physical environment questionnaire from KCHS. However, many of the questions and items used in our study have been used in previous nationwide studies or have been validated by previous studies. Because social resources are culture- and context-dependent, different Korean versions of the instrument need to be developed and validated for different regional populations in the future [30]. Also, data were collected at the individual level, so it is not possible to make direct claims about population-level effects and the results more prone to endogeneity because health status or confounding variables are more likely to influence individual-level assessments than area-level assessments.

Second, although our population samples are representative in Korea, this study used cross-sectional data from 2013, and therefore there are limits in generalizing the observed relationships. Thus, there is a need to confirm our findings in further studies across different time periods, with prospective cohort study over time. 

Third, we did not conduct studies pertaining to social network services, such as Facebook and Twitter, which contribute to personal networks, social support, or social participation and activities in today’s society, but these elements should be added in future studies. 

Fourth, we could not include other possible confounding factors, such as disease condition or treatment, because the national survey dataset does not provide that information. 

Lastly, Because of with a large sample size, a small change could show effects that are statistically significant in this study. Henceforth, the minimum social or public health programs or policies important difference should be defined a prior and the sample size determined accordingly.

Despite these limitations, these results add to the body of literature examining the relationships between perceived social and physical environments and SRH in different residential areas. Although individual-level analyses are inherently limited to make conclusions about share characteristics or conditions, the current study could be used to understand urban and rural differences in the associations between social and physical environments and health status through comparison with previous studies conducted in other Asian countries. There should be further study reflected Korean culture or contextual effects, which is covered by aggregation of individual scores analyses.

## 5. Conclusions

The present study focuses on individual-level social and physical environments and self-rated health in Korean adults living in both urban and rural areas. The main findings of our study are the positive associations between social and physical environmental variables (trust of neighbors, exchange of help with neighbors, participation in social activities or organizations, and satisfaction with physical environments) and perceived health in both urban and rural areas (although in rural areas, there is an inverse association between longer residence and poor health after covariate adjustment). Also, frequency of contact with relatives and SRH did not appear to impact health in either rural or urban areas, whereas more contact with neighbors was positively associated with good health in rural, but not urban, areas.
